# Communication between secondary and primary care following self-harm: are National Institute of Clinical Excellence (NICE) guidelines being met?

**DOI:** 10.1186/1744-859X-7-21

**Published:** 2008-10-23

**Authors:** Jayne Cooper, Elizabeth Murphy, Rita Jordan, Kevin Mackway-Jones

**Affiliations:** 1 Community Based Medicine, Department of Psychiatry, University of Manchester, UK; 2 Manchester Mental Health and Social Care Trust, Manchester, UK; 3 Emergency Medicine Research Group, Manchester Royal Infirmary, Manchester, UK

## Abstract

**Background:**

Most patients contact their general practitioner (GP) following presentation to an Emergency Department (ED) after a self-harm incident, and strategies to help GPs manage these patients include efficient communication between services. The aim of this study was to assess the standard of documentation and communication to primary care from secondary care as recommended by the National Institute of Clinical Excellence (NICE) guidelines on the short-term management of people who self-harm.

**Methods:**

An audit of medical records (ED and Psychiatric) on people aged 16 years and over who had presented to the ED following self-harm, benchmarked according to government guidelines, was performed. Data were collected over a 4-week period at a general teaching hospital.

**Results:**

We collected data on 93 consecutive episodes of self-harm; 62% of episodes were communicated to primary care, 58% of these communications were within 24 h and most within 3 days. Patient identifying details and follow-up arrangements were specified in most cases. Communication via psychiatric staff was most detailed. ED clinicians provided few communications and were of limited content. Communication with the patient's GP was not made in half of those cases seen by a mental health specialist.

**Conclusion:**

Government guidelines are only partially being met. Reliance on communication by ED staff would leave a substantial proportion of patients discharged from the ED with no or minimal communication to primary care. Psychiatric services need to improve the rate of communication to the patient's GP following assessment A national sample of National Health Service (NHS) trusts would establish if this is a problem elsewhere.

## Background

General Practitioners (GPs) are pivotal in the management of patients who self-harm. A recent review [1] suggested that one strategy that helps GPs manage these patients is efficiency of communication between secondary and primary care. Most patients who present to the Emergency Department (ED) with self-harm consult their GP soon afterwards [2,3]. Many of these patients present with physical complaints, which may disguise an underlying risk of psychological problems [4]. At first point of contact after an episode of self-harm, the patient may be unknown to the GP. Prior knowledge of this history should assist in their assessment, yet less than half (41%) of GPs receive a discharge summary [5].

Clinical practice guidelines are systematically-developed statements that aim to assist physicians in supplying the appropriate health care for specific clinical circumstances [6]. Government guidelines in the UK provide recommendations for practice based on the best available evidence. One such guideline on the short-term management of self-harm patients in secondary care recommends that all patients presenting with self-harm should receive a psychosocial needs assessment including social, psychological and motivational factors, and mood and risk [7]. This information should be passed on to GP 'as soon as possible'. Mental capacity and evidence of mental illness should be communicated to GPs if patients leave before a specialist assessment. The guidelines endorse the Royal College of Psychiatrists' (RCP) recommendations [8] on the content of these communications. However, implementation of National Institute of Clinical Excellence (NICE) guidelines has been variable [9].

In light of developments in national policies and further research, the Royal College of Psychiatrists produced a revision of their earlier document [10] on a consensus of standards for assessment following self-harm. Several standard competencies (for non-specialist staff and specialist staff) were recommended [8]: psychosocial assessments should ideally be carried out by specialist staff; medical staff may undertake assessments if trained for the task; where a patient is not admitted or assessed by a specialist, ED staff should record information according to the recommended list below, which should be passed onto the GP; there should be a policy for referral to specialists for patients admitted to general medical wards; and information from assessments should be passed on to the GP (by fax) within 24 hrs and written communication within 3 working days. The recommended patient information to be obtained before discharge following self-harm included specific arrangements for any follow-up if not referred on for specialist opinion.

The aims of the present study were to assess the standard of documentation and communication after presentation to an ED in the northwest of England with self-harm of patients aged 16 years and over, based on national guidelines. Our objectives were to assess: the proportion of self-harm cases that were discharged from EDs that were notified to primary care services; the content and nature of communications from EDs to primary care services about such patients; the proportion of self-harm cases that were assessed by psychiatric services that were notified to primary care services; the content and nature of communications from psychiatric services to primary care services about such patients; and the timing of communication between EDs/psychiatric services to primary care services.

### Definition of self-harm

Self-harm is defined as intentional self-poisoning or self-injury, irrespective of motivation [11]. Self-poisoning includes the intentional self-ingestion of more than the prescribed amount of any drug, whether or not there is evidence that the act was intended to result in death. This also includes poisoning with non-ingestible substances and gases, overdoses of 'recreational drugs' and severe alcohol intoxication where clinical staff considers such cases to be an act of intentional self-harm (rather than recreational binge drinking). Self-injury is defined as any injury that has been intentionally self-inflicted.

### Service configuration

Service configuration at the study hospital included psychiatric nurse led ED liaison team (operating 7 days a week from 9 am to 10 pm). The on-call junior doctors in psychiatry are available 24 h a day. In addition the Self-Harm, Assessment, Follow-up and Engagement (SAFE) Team assess people who present with self-harm and live within the catchment area and are registered with a GP. GPs may receive correspondence based on ED records if patients have not been assessed by psychiatric services and are out of area. Within the ED, it is incumbent upon the doctor to make appropriate arrangements for follow-up.

## Methods

This was a prospective case note study using ED/medical/mental health records in a general teaching hospital in the northwest of England, UK. All episodes of self-harm of patients 16 years and over between 18 January and 14 February 2006 were identified using information from a monitoring database of self-harm known as the Manchester Self-Harm (MaSH) project [12]. We completed a proforma on all episodes. The proforma was based on criterion derived from NICE guidelines and RCP recommendations.

## Results

During the 4-week period there were 93 episodes of self-harm, 40% male, 60% female (aged between 16–73 years). In total there was evidence of communication to the patient's GP in 58/93 (62%) of cases. The source of these communications included 26 from psychiatric staff only, 3 from ED staff only, 3 from both psychiatric staff and ED staff and 26 from the SAFE liaison service. The speed of communications (percentage of all communications) was 34/58 (59%) of episodes within 24 h, 19/58 (33%) within 3 days and 5/58 (9%) after 3 days. The method of communication (percentage of the total 58 communications) was predominantly by fax with 52/58 (90%), 3/58 (5%) by letter and in 3/58 (5%) the method was not recorded. Table 1 provides a breakdown of the content of these communications and by discipline.

**Table 1:** Content of communications from secondary services to primary care services according to recommendations by Royal College of Psychiatrists (2004)

**Type of information**	**Any communication, including SAFE liaison follow-up ** **(n = 58)**	**Emergency Department staff only** **(n = 3)**	**Psychiatric services** **(n = 29)**	**SAFE liaison follow-up letter** **(n = 26)**
Name/age/date of birth	58/58	3/3	29/29	26/26

Past psychiatric history	23/58	0/3	23/29	0/26

Past history of self-harm	8/58	0/3	8/29	0/26

Comment on mood	22/58	0/3	21/29	1/26

Reference to alcohol	22/58	0/3	11/29	11/26

Reference to drugs	3/58	0/3	3/29	0/26

Precipitating circumstances	26/58	0/3	22/29	4/26

Follow-up arrangements	57/58	3/3	29/29	25/26

SAFE, Self-Harm, Assessment, Follow-up and Engagement.

### Communications by ED staff

ED staff completed a psychosocial assessment on 47/93 (51%) of episodes. In 74% of episodes, patients were either admitted to medical ward and/or seen by Psychiatric services, with no follow-up in 26% of episodes. Of those without follow-up, ED staff communicated with the patient's GP in 13% of episodes and all communications were made within 24 h.

### Communications by psychiatric staff (not including SAFE liaison service)

Psychiatric staff assessed 56/93 (60%) episodes, and 29/56 (52%) of these assessments were communicated to the GP. Duty doctors were responsible for a large proportion of assessments (43/56, 77%) and 19/43 (44%) were communicated to the GP. Liaison nurses communicated 5/8 (63%) and SAFE team 5/5 (100%) of their assessments.

### Communications by SAFE liaison service

**Figure 1:**
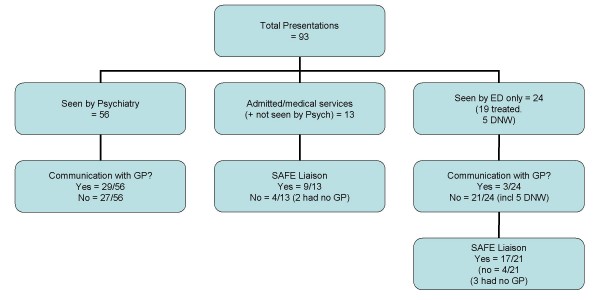
Communication with the General Practitioner by service provision pathway.

The psychiatric service configuration at the study hospital however enabled a further 26 episodes to be communicated to the GP based on information gleaned from ED records (SAFE Liaison service). Figure 1 illustrates the service provision pathway and communication to primary care following an episode of self-harm.

## Conclusion

Nearly all self-harm episodes at the study hospital were either seen by specialist psychiatric staff, or a member of the SAFE team would write to the patients' GP, if registered, using information available from ED records. However, for those individuals not seen and assessed by specialist staff this information was limited. Communication with the patient's GP was not made in half of those cases seen by a mental health specialist. We suggest that psychiatric services (particularly doctors as they appear to assess proportionately more patients) need to improve the rate of communication to the patient's GP following assessment.

Although the SAFE team provide a liaison or "add-on communication service" for those not seen by a specialist, they cannot compensate for the lack of information recorded by ED staff. Reliance on communication by ED staff would leave a substantial proportion of patients discharged from the ED with no or minimal communication to primary care. A national sample of NHS trusts would establish if this is a problem elsewhere. EDs must have procedures in place to aid communication between the psychiatric service and primary care if discharge directly from the ED. We suggest that ED staff protocol might include completion of an initial psychosocial assessment using a proforma designed for the task, which could be forwarded directly to primary care.

Due to increased specialization of service provision and subsequent criteria for meeting inclusion, patients remain liable to fall between service boundaries. This leaves an important role for GPs. External validity of the study is limited to EDs in urban areas with similar service provision and variability in organisations and provision of service for this group has been reported in the UK [13]. The study demonstrates that even with a bespoke self-harm service these patients can fall between service provisions. This emphasises the role of ED staff in the subsequent psychosocial management of self-harm patients.

## Competing interests

The authors declare that they have no competing interests.

## Authors' contributions

JC and KM-J, designed the original study. RJ collected the data, and EM and JC analyzed and interpreted the data. JC wrote the article with contributions from EM, RJ and KM-J. All authors read and approved the final manuscript.
